# 

**Published:** 1885-08

**Authors:** 


					﻿JVCJG -TJST, 1885.
J.____________________________________________________________
L >
O
: SOLD SEBAfefr-
xJp	Vol. XXV.

(^og^PTI
e NEW SERIES
*	-' \/„l	11	CJ
|4|
IjR (JOURNAL®.
fex et|p /	\Wfoiore •>
J»j	V i>t-<?>
-p by" W.F.WESTMOREtANDJ®
gk h.v.m.miller.m.d.ll.dJs<
p-''	JAMES.A.GRAY. M.D.
wiblished By james.p.harrison&cqU
iff_______________«flTLANTA,GA. jfiM
SUBSCRIPTION : S2.5O A YEAR.
				

